# Investigation of sinonasal anatomy via low-dose multidetector CT examination in chronic rhinosinusitis patients with higher risk for perioperative complications

**DOI:** 10.1007/s00405-016-4268-y

**Published:** 2016-08-23

**Authors:** Marcin Fraczek, Maciej Guzinski, Monika Morawska-Kochman, Tomasz Krecicki

**Affiliations:** 1 Department of Otolaryngology, Wroclaw Medical University, 213 Borowska Str, 50-556 Wrocław, Poland; 2 Department of Radiology, Wroclaw Medical University, 213 Borowska Str, 50-556 Wrocław, Poland

**Keywords:** Rhinosinusitis, Nasal polyps, Computed tomography, Low dose, Radiation, Complications, Anatomy, Endoscopic sinus surgery

## Abstract

The aim of the study was to compare visualisation of the surgically relevant anatomical structures via low- and standard-dose multidetector CT protocol in patients with chronic rhinosinusitis (CRS) and higher risk for perioperative complications (i.e. presence of bronchial asthma, history of sinus surgery and advanced nasal polyposis). 135 adult CRS patients were divided randomly into standard-dose (120 kVp, 100 mAs) or low-dose CT groups (120 kVp, 45 mAs). The detectability of the vital anatomical structures (anterior ethmoid artery, optic nerve, cribriform plate and lamina papyracea) was scored using a five-point scale (from excellent to unacceptable) by a radiologist and sinus surgeon. Polyp sizes were quantified endoscopically according to the Lildholdt’s scale (LS). Olfactory function was tested with the “Sniffin’ Sticks” test. On the low-dose CT images, detectability ranged from 2.42 (better than poor) for cribriform plate among anosmic cases to 4.11 (better than good) for lamina papyracea in cases without nasal polyps. Identification of lamina papyracea on low-dose scans was significantly worse in each group and the same was the case with cribriform plates in patients with advanced polyposis and anosmia. Cribriform plates were the most poorly identified (between poor and average) among all the structures on low-dose images. Identification of anterior ethmoid artery (AEA) with reduced dose was insignificantly worse than with standard-dose examination. The AEA was scored as an average-defined structure and was the second weakest visualised. In conclusion, preoperatively, low-dose protocols may not sufficiently visualise the surgically relevant anatomical structures in patients with CRS and bronchial asthma, advanced nasal polyps (LS > 2) and history of sinus surgery. Low mAs value enables comparable detectability of sinonasal landmarks with standard-dose protocols in patients without analysed risk factors. In the context of planned surgery, the current preferences of the tube should be carefully evaluated for different patient constitutions to minimise the risk of complications.

## Introduction

Endoscopic sinus surgery (ESS) is considered to be an effective treatment modality of choice for patients suffering from chronic rhinosinusitis (CRS) who fail medical therapy. Adequate preoperative patient evaluation involves computed tomography (CT) examination, which is paramount in preventing complications in ESS [1]. The growing prevalence of patients complaining of sinus-like symptoms requiring diagnostic imaging has increased the awareness of potential hazards from radiation exposure. Consequently, several attempts were made to elaborate the reduced-dose CT protocols which are now often employed as a screening method for the detection of inflammation within sinuses. Although reducing the radiation dose is accompanied by compromised CT image quality, low-dose scanning has come to be increasingly applied for preoperative planning. This is probably because ESS is often considered to be a relatively safe procedure and due to the growing number of easily accessible low-dose CT scanners. This is despite the fact that the appropriateness of dose reduction in pre-endoscopic examination has not been established and given that some reports suggest for that reason only standard-dose (SD) protocol [2, 3]. There is still a lack of both universally accepted standards of disease-related image quality and reliable comparisons of the visualisation of surgically relevant anatomical structures on low- and standard-dose CT images.

Congenital anomalies, anatomical variants and post-surgical changes undetected on preoperative CT scans can lead to pathological consequences due to the proximity of the sinonasal region to vital structures. It has been shown that the standard-dose sinus CT provides a high (about 90 %) consistency of radiological and intraoperative findings [4]. A similar level of consistency has not yet been analysed in relation to low-dose CT scanning.

It could be suspected that using dose reduction CT protocol in chronic rhinosinusitis patients with initially higher risk for perioperative complications, like those with bronchial asthma, after previous sinus surgery and with enhanced nasal polyposis, may potentially further increase the risk of complications [5–7].

The aim of the study was to assess the extent to which the use of a low-dose multidetector CT protocol affects the identification of surgically relevant anatomical structures in patients with chronic rhinosinusitis and the above-mentioned risk indicators. Therefore, this analysis is also an attempt to customise radiation doses to the clinical indication and the patient’s constitution.

## Materials and methods

### Patient selection

A cross-sectional, uncontrolled, randomised, single-blind study was conducted on symptomatic, uncomplicated adult patients with CRS admitted to the Department of Otolaryngology at Wroclaw Medical University. CRS was diagnosed according to previously established rules [8]. The patients were selected consecutively as they fulfilled the criteria for participation in a prospective study. Paranasal sinus imaging was part of the routine preoperative clinical assessment. CT scans were scored according to the Lund–MacKay (L–M) staging system (ranging from 0 to 24). Patients without bilateral inflammatory changes were excluded. Diagnosis of asthma was confirmed by pulmonary function testing according to the GINA [9]. The ASA intolerance was confirmed by aspirin challenge [10]. Each patient gave written consent for the CT examination to be performed and to participate in the study. The study protocol was approved by our institutional review board.

### Endoscopic appearance scores

Polyp sizes were quantified endoscopically according to the Lildholdt’s scale (LS) which classifies nasal polyps into four stages: no polyposis (0), polyps only in the middle meatus (1), polyps beneath the lower edges of the middle turbinate (2) and large polyps reaching the lower border of the inferior turbinate (3) [11]. The total score might range from 0 to 6.

### Assessment of olfactory function

Olfactory function was tested with the Sniffin’ Sticks Screening 12 Test (Burghart, Wedel, Germany) according to manufacturer’s instruction and previous report [12]. The final score classifies subjects into three groups: normosmic, hyposmic and anosmic.

### Computed tomography examination

CT examination of all the patients was performed on a GE Discovery 750 HD scanner (General Electric Healthcare, Milwaukee, USA). Low-dose protocols included parameters formulated on a cadaver head: tube potential 120 kVp, 45 mAs; detector configuration 64 × 0.625 mm; pitch 1.3; section thickness 0.625; and gantry rotation time 0.4 s [3]. The images were reconstructed with an adaptive statistical iterative reconstruction (ASIR) algorithm using 50 % ASIR.

In the standard-dose group, the following protocol was used: tube potential 120 kVp, 100 mAs; detector configuration 64 × 0.625 mm; pitch 0.9; section thickness 0.625; gantry rotation time 0.7 s. All other scanning parameters were the same for both groups. Patients were randomly selected for LD or SD protocols according to the day of examination. LD was performed on Mondays, Wednesdays and Fridays, whereas SD was performed on Tuesdays and Thursdays. During the experiment, participants were blind to the doses.

### Subjective evaluation of image quality

Quantitative analysis was performed for each study using certified diagnostic workstations: AW 4.6 (GE Healthcare, USA). The reviewed images were reconstructed with 0.625 slice thickness and displayed with a window level/width of 2000/350 HU and sharp filter-s3. Two researchers, a radiologist (MG) and an ENT specialist (MF) (both with 11 years of experience), evaluated the reformatted axial and coronal images of each patient independently and separately in a blind manner.

Using a five-point subjective scale [5-excellent image quality, 4-good, 3-moderate (hardly any artefacts), 2-poor (many artefacts), 1-unacceptable], four surgically relevant anatomical structures were assessed: the course of the anterior branch of the ethmoidal artery (AEA), the course of the optical nerve (cranial nerve II; CNII) next to the sphenoid sinus, cribriform plate (CP) and lamina papyracea (LP). The ratings of both researchers were averaged for each structure.

### Estimation of radiation doses

Radiation dose descriptors were derived from the dose report automatically stored in Picture Archiving and Communication Systems. CT dose index volume (CTDIvol), dose length product (DLP) and scan range were recorded for both the LD and SD groups. To calculate the effective doses (mSv), the DLP was multiplied by region-specific normalised effective dose conversion coefficient (*k*) for the head [*k* = 0.0023 mSv/(mGy/cm)], as proposed by the European Working Group for Guidelines on Quality Criteria in CT [13].

### Statistical analysis

Statistical computations were performed using Statistica PL software package version 10.0. The two-sided *t* test with independent variables was used as the test of significance for the analysis of the image quality. For all analyses, we considered the 95 % confidence interval and 5 % significance interval (*p* < 0.05).

## Results

### Clinical data of the studied patients and radiation doses

Clinical data for the 135 patients enrolled into the low- and standard-dose groups are shown in Table 1. There were no significant differences between the two groups in terms of patient age, gender distribution, advancement of inflammation in sinuses on CT scans (L–M scale) and endoscopic score according to LS. There were slightly more patients with a history of sinus surgery in the SD group.

**Table 1:** Comparison between chronic rhinosinusitis patients diagnosed with low- or standard-dose multidetector CT protocols

Patient characteristics	Low-dose protocol (*n* = 83)	Standard-dose protocol (*n* = 52)
Female/male, *n*	44/39	29/23
Mean age (years)	50.4	49.3
DLP (mGycm), mean (S.D.)	34.12 (6.53)	341.36 (58.62)*
Lund–MacKay CT score, mean (S.D.)	10.28 (6.75)	11.4 (6.02)
Lildholdt’s scale, mean (S.D.)	1.56 (1.77)	2.08 (1.72)
0 acc. Lildholdt’s scale, *n* (%)	38 (46)	16 (31)
1–2 acc. Lildholdt’s scale, *n* (%)	17 (20)	18 (34.5)
>2 acc. Lildholdt’s scale, *n* (%)	28 (34)	18 (34.5)
Bronchial asthma, *n* (%)	16 (19)	11 (21)
History of sinus surgery, *n* (%)	18 (22)	22 (42)
Anosmic, *n* (%)	22 (26)	16 (30)

* *p* < 0.001

Both, the group of patients with anosmia and asthma were relatively homogeneous. Each patient in these groups had polyps on nasal endoscopy. However, two (9 %) of the anosmics in LD and four (25 %) in the SD group had nasal polyposis graded <3 according to LS. Similarly, there was one (6 %) asthmatic with small nasal polyps (LS < 3) in the LD group and three (27 %) similar cases in the SD group.

The mean value of the effective radiation dose for the SD CT protocol was 0.785 mSv, which was ten times higher than that of the LD protocol (0.078 mSv).

### The accuracy of sinus anatomy visualisation in patients with brachial asthma

**Fig. 1:**
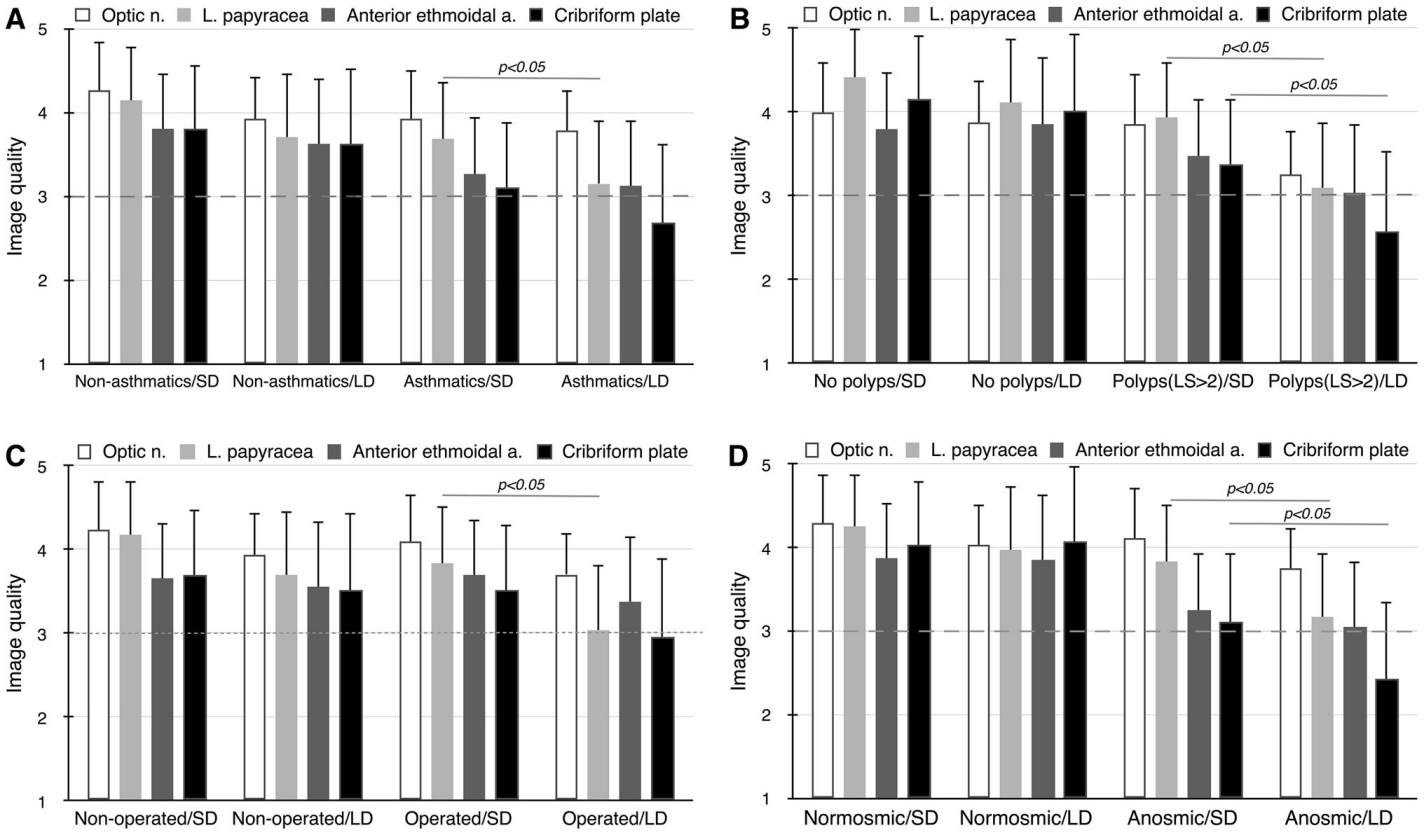
The accuracy of sinus anatomy visualisation (mean ± S.D.) in patients with brachial asthma (**a**), enhanced nasal polyposis (>2 acc. Lildholdt’s scale) (**b**), history of sinus surgery and anosmia (**d**) via low-dose (LD) and standard-dose (SD) multidetector CT protocols. Image quality ranged from 1 (unacceptable) to 5 (excellent). *Dashed line* indicates threshold of acceptability. Statistical image quality deterioration compared to the standard-dose examination

In non-asthmatics with CRS, image quality ranged from 3.62 for AEA and CP to 3.93 for CNII using an LD CT protocol and from 3.8 for CP to 4.26 for CNII in an SD examination (Fig. 1a). The differences between protocols were insignificant.

In asthmatics, the ability to identify the anatomy ranged from 2.69 for CP to 3.78 for CNII at LD and from 3.12 for CP to 3.92 for CNII in standard-dose imaging. The LD compared to SD protocol worsened the detectability of each anatomical structure, but only for lamina papyracea in a significant manner (3.16 vs. 3.69; *p* < 0.05). The sinus surgeon scored the visualisation of CP, LP and AEA on LD scans below moderate levels (2.44, 2.94 and 2.94, respectively) which had previously been set to represent minimum acceptable image quality [3].

### The ability to define sinus anatomy in patients with nasal polyps

In patients without polyps, detectability of anatomical structures ranged from 3.86 for AEA to 4.11 for LP on LD CT scans (Fig. 1b). SD CT protocols allowed insignificantly better visualisation with image quality ranging from 3.87 for AEA to 4.42 for LP.

In a group of patients with enhanced polyposis (LS > 2), image quality ranged from 2.58 for CP to 3.8 for LP on LD scans, and from 3.36 for CP to 4.11 for CNII on SD CT scans. Identification of anatomical details was better on SD images and achieved statistical significance for LP (3.1 vs. 3.94) and CP (2.58 vs. 3.36) (*p* < 0.05). The sinus surgeon rated visualisation of CP, AEA and LP on LD CT scans below average levels (2.28, 2.84 and 2.88, respectively).

### The ability to visualise sinus anatomy among patients with history of sinus surgery

Among unoperated CRS patients diagnosed with reduced dose, visualisation of anatomy was in the range from 3.52 for CP to 3.94 for CNII (Fig. 1c). SD protocols delivered insignificantly better scans, rated from 3.66 for AEA to 4.24 for CNII.

In subjects with previous sinus surgery, SD CT examination visualised the structures at levels from 3.52 for CP to 4.09 for CNII. Dose reduction techniques led to a deterioration in the definition of the anatomy to a range from 2.96 for CP to 3.71 for CNII. The difference between LD and SD protocols reached statistical significance only with respect to LP (3.04 vs. 3.84; *p* < 0.05). In that group, the sinus surgeon scored the ability to identify CP on LD scans below average levels (2.83).

### The accuracy of sinus anatomy visualisation among anosmic patients

Both SD and LD examinations enabled similar identification of all anatomical structures in patients with CRS and normal smell (Fig. 1d).

**Fig. 2:**
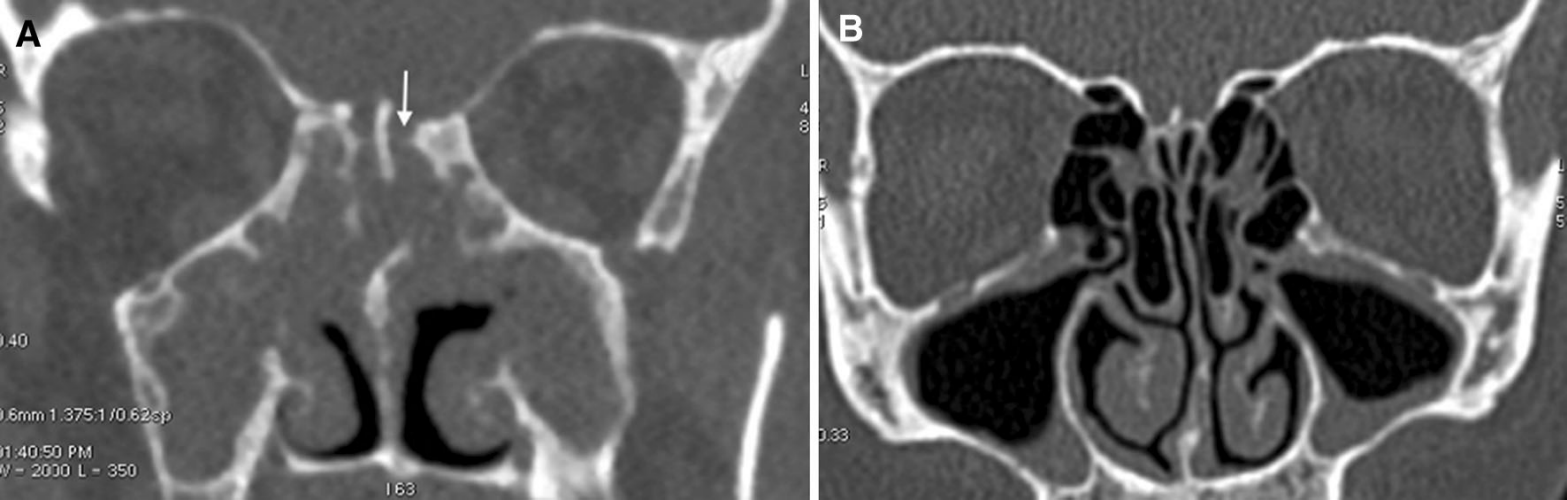
Coronal images of multidetector CT scans obtained according to low-dose (120 kVp, 45 mAs) protocol in patients with chronic rhinosinusitis. Significant image quality deterioration (**a**) seen i.a. in olfactory fossa (*arrow*) in anosmic subject with massive nasal polyposis (>2 acc. Lildholdt’s scale). Well-defined bony margins (**b**) in normosmic patient without polyps in nasal endoscopy

In anosmic subjects with CRS diagnosed with SD CT, anatomy was identified at levels from 3.12 for CP to 4.12 for CNII. The use of LD compared to SD CT led to a significant deterioration in the detectability of LP (3.18 vs. 3.84; *p* < 0.05) and CP (2.42 vs. 3.12; *p* < 0.05). The sinus surgeon scored the ability to identify CP, AEA and LP on LD scans below average levels (2.2, 2.9 and 2.95, respectively) (Fig. 2).

There were no significant correlations in the groups of patients with hyposmia and polyps graded 1–2 according to Lildholdt’s scale (data not shown).

## Discussion

Endoscopic sinus surgery is often classified as a potentially high-risk procedure. Although the overall incidence of major complications is low (0–2 %), minor complications occur in up to 20 % of patients [14, 15]. The clear identification of key landmarks in this region preoperatively on CT scans minimises the risk for intra- and postoperative difficulties. The present study includes radiological identification of vital anatomical structures, the failure of which may cause the most common complications during ESS, i.e. haemorrhage, cerebrospinal fluid (CSF) leakage and orbital injury [6, 16]. The described low-dose CT protocol enabled identification of anatomy scored from 2.42 (better than poor) for cribriform plate among anosmic patients to 4.11 (better than good) for lamina papyracea in cases without nasal polyps.

Lamina papyracea was the only structure for which visibility in each of the analysed groups (i.e. among asthmatics, patients with enhanced polyposis, anosmia and history of sinus surgery) was significantly worse on low-dose than on standard-dose CT scans. Thinning and congenital or acquired defects of the LP which increase the risk of orbital damage can easily be overlooked on standard-dose CT scans and are unlikely to be identified with reduced-dose examination in the higher-risk patients, as suggested by the present data. Another consequence of insufficient pre-endoscopic visualisation of LP and the skull base is the possible limitation of the surgery resulting from greater caution on the part of the surgeon. Residual, unopened ethmoid cells at the LP are a common finding in revision ESS, even in as many as 79 % cases [17]. Simultaneously, recent studies suggest extending ESS range in subjects with nasal polyps and asthma to prolong the time interval until revision surgery [18].

Definition of the cribriform plate was only significantly worse on low-dose scans in subjects with advanced polyposis and lack of smell. However simultaneously, CP was the poorest identified among all the important structures. The lateral lamella of the CP might only be 0.05 mm and this is the weakest part of the skull base and a common site of CSF leak [19]. An important finding during preoperative review of CT is to note the length of the lateral lamella and asymmetry of the CP, which is observed in 50 % of cases [20]. The incidences of CSF leak during endoscopic sinus surgery are mostly the result of misjudging the anatomy. Hypothetically, this might happen more frequently in subjects with the above risk factors diagnosed with low-dose CT technique.

The detectability of the anterior ethmoid artery by reduced-dose protocols was revealed not to be significantly worse compared to standard-dose examinations. However, the AEA was the second most poorly defined anatomical structure after CP, in particular, in the opinion of the sinus surgeon. The AEA can be easily injured during surgery, which is the most frequent cause of orbital haematoma. In 40 % of cases, the artery has a separate osseous canal at a distance of approximately 3.5 mm from the skull base [21]. Present data suggest that low-dose CT examination in some cases may not be sufficient to determine AEA location.

The optic nerve was the most visible among the analysed anatomical structures on low-dose scans independent of the group of patients. This may be explained by the relatively large nerve diameter, 0.3 mm thick, and rarely dehiscent (less than 8 %) bone coverage [22, 23].

The obtained data show that the changes resulting from previous sinus surgeries including altered anatomy, scarring and neo-osteogenesis cause a greater deterioration in image quality on low-dose scans than on standard-dose CT scans. A significant difference between protocols was found only for lamina papyracea, but identification of cribriform plate on low-dose images in reoperated subjects was also unsatisfactory. A careful study of reduced-dose CT scans appears to be particularly important in patients before revision surgery who are considered to have an increased risk of complications [6].

Both severe polyposis and loss of smell are the consequences of advanced inflammatory processes within sinuses. Similarly, coexistence of bronchial asthma and rhinosinusitis often results in enhanced nasal polyps. One could, therefore, expect convergent results in the evaluated groups of patients. A separate analysis enabled proof of the predictive value of each of the clinical parameter. On the basis of medical history (presence of anosmia, asthma or history of sinus surgery) or nasal endoscopy (detection of sever polyposis), it can be assessed whether the definition of anatomical structures will be significantly deteriorated on low-dose scans. Although anosmia is not a risk factor for intraoperative complications, it correlates well with the severity of the disease and is easy to evaluate. Previous data revealed that the self-rated olfaction function correlates significantly with measured olfactory function [24].

Both, patients with asthma and anosmia had polyps in nasal endoscopy. Subjects with polyps graded <3 according to Lildholdt’s scale presented high degree of sinuses opacification on CT scans (L–M > 12) which influenced the image quality, and thus these cases were not excluded from the study. In future, it would be interesting, however, to examine in the similar manner asthmatics without nasal polyps.

Optimisation of the radiation doses is an important issue in diagnostic radiology. Consequently, low-dose protocols are more widely used and have replaced standard settings recommended by the CT manufacturers. According to opponents, a dose of 6–14 Gy required for cataract formation would be absorbed after 100–200 standard CT examinations, which is much more than any patient is likely to receive [25]. The risk of radiation-induced malignant neoplasms of the thyroid based on a stochastic risk is stated to be 0.0075 per Gy [26]. This means that the likelihood of inducing a cataract or thyroid cancer after being examined with even periodically repeated standard CT scanning seems to be negligible.

Finally, an important consideration is the medico-legal consequence of a doctor’s decisions. In this regard, it should be remembered that patients being treated for rhinosinusitis account for about 70 % of all malpractice claims against otolaryngologists [27].

The incidence of complications during ESS in patients with preoperative low-dose and standard-dose protocols has not been compared yet. In the present analysis, there was only one case of palpebral ecchymosis (data not shown) in each of the groups, but the number of patients included in the study did not enable reliable conclusions to be drawn. This is, to a certain degree, a limitation of the work.

Exposure parameters used in the present comparison were chosen on the basis of our previous study on a cadaver head defining the minimum acceptable image quality and institutional ALARA (As Low As Reasonably Achievable) protocol [3]. Radiation dose for the described technique is lower than for the cone beam computed tomography (CBCT) considering the appropriate field of view (0.078 vs. 0.2 mSv, respectively) [28]. The above settings correspond with results from previous reports recommending scanning at 40–60 mAs for confirmation of the clinical diagnosis of sinusitis [29–31]. According to some studies, such parameters reduce the radiation significantly while retaining an image quality sufficient for assessing the osseous structures [31, 32]. In contrast to the present work, the major limitation of those researches was the use of only phantoms, cadaveric heads and the lack of clear inclusion criteria, even if the study was performed on patients.

In conclusion, the low-dose technique has a reasonable diagnostic value for screening chronic rhinosinusitis. However, in the context of planned surgical interventions, when knowledge of the individual anatomy becomes particularly important, its application should be well thought out. Higher mAs settings enable a more accurate evaluation of surgically relevant anatomical structures and should be considered, especially among those subjects with an initially higher chance of intraoperative difficulties. CT scans should alert the surgeon to the potential for injury, but it seems that low-dose protocols may not do this sufficiently well in patients with bronchial asthma, advanced nasal polyps, history of sinus surgery or anosmia. In all other uncomplicated cases, deterioration of image quality as a result of dose reduction is minor and should not preclude its use. When choosing an appropriate preoperative scanning protocol, it is worth recalling the maxim “The eyes see what the mind knows”, which is very true in the case of endoscopic sinus surgery.
